# Efficacy of CO_2_-baited mosquito catchers in controlling vector mosquitoes in residential areas of China

**DOI:** 10.3389/fvets.2025.1671416

**Published:** 2025-10-02

**Authors:** Yuyan Wu, Chuan Zhang, Yanyang Peng, Jimin Sun, Zhenyu Gong, Feng Ling

**Affiliations:** ^1^Zhejiang Provincial Center for Disease Control and Prevention, Hangzhou, Zhejiang, China; ^2^Fenghua District Center for Disease Control and Prevention, Ningbo, Zhejiang, China

**Keywords:** vector control, CO₂-baited trap, *Aedes albopictus*, residential mosquito management, breathing mosquito catcher

## Abstract

**Introduction:**

Vector-borne diseases significantly impact global health. Mosquitoes are key vectors for transmitting such diseases, making mosquito control crucial for disease prevention. Carbon dioxide is commonly used in surveillance traps to attract mosquitoes. However, its application in mosquito abatement devices is limited due to environmental and logistical constraints related to continuous CO_2_ emissions. In China, a new mosquito trap utilizing CO_2_ was developed using nanoporous silicon-based polymer materials to capture and release CO_2_ from the air. This study aimed to assess the operational efficiency of this CO_2_-enhanced trap in reducing mosquito populations and its potential for residential vector control applications.

**Methods:**

Two residential villages with similar mosquito densities and geographic environments were selected for field trials in Ningbo City in 2024. One village was randomly assigned as the control group, while the other served as the test group. Within the test group, three zones were artificially divided to evaluate the effect and scope of the breath-activated mosquito trap in managing mosquito populations. Adult and larval mosquito densities were monitored bi-monthly before and after the trials using BG traps, CDC light traps, and the larval pipette method.

**Results:**

Larval and adult mosquito densities were monitored for four months before the trials to establish the baseline mosquito density between the test and control villages; no statistical differences were found (larval, U = 35, *p* = 0.798 > 0.05; adults, CDC light trap U = 41, *p* = 0.442 > 0.05, BG trap U = 43, *p* = 0.279 > 0.05). After the trials began, standard decreasing rates of larval and adult mosquito densities were observed, with 36.24%–46.93% larval mosquito density decreasing, 38.22%–65.91% (CDC light traps), and 43.05%–73.30%; BG traps. Statistically significant differences were found between Zone I and the control village for larval and adult mosquitoes (GLMM, larval *p* = 0.026; adult CDC light trap *p* = 0.009; BG trap *p* = 0.027).

**Discussion:**

Breathing catchers using CO_2_ can effectively control larval and adult mosquito densities in a range of regions. Without insecticide usage and excess CO_2_ emissions, this might be an effective choice for mosquito control in residential areas to prevent mosquito-borne diseases.

## 1 Introduction

Vector-borne diseases have become significant contributors to global morbidity and mortality, presenting a substantial public health challenge. The World Health Organization (WHO) estimates that approximately 700,000 deaths occur annually due to these diseases (1). Mosquito-borne diseases, such as malaria, chikungunya, dengue, yellow fever, and Zika virus disease, are widespread and severe. In 2018, malaria alone resulted in around 405,000 deaths, while dengue cases have surged 15-fold since 2000, impacting over 129 countries globally (1).

Mosquitoes are among the most crucial vectors of mosquito-borne diseases. In China, the mosquito fauna is diverse, comprising approximately 390 species, including the genera *Aedes*, *Culex*, and *Anopheles*. These species serve as potential vectors for a range of diseases, such as dengue, chikungunya, Zika virus infections, Saint Louis encephalitis, West Nile fever, and malaria (2–4). Global warming has led to the expansion of the distribution range of some mosquitoes, such as *Aedes albopictus*, in China, putting over 168 million individuals at high risk of dengue infection annually (5, 6). Reports suggest that the estimated incidence rate of dengue fever in China is 0.04 per 100,000 people (2). Given the lack of effective vaccines and commercial drugs, mosquito control is crucial for curbing the spread of mosquito-borne diseases.

In recent decades, various methods to attract and kill mosquitoes have gained attention, including attractive toxic sugar baits (ATSB), specific-wavelength ultraviolet light, diverse commercial lure attractants, and CO_2_. ATSB, developed based on mosquitoes’ feeding behavior, effectively reduces mosquito density in both laboratory and field settings (7, 8). However, incorporating chemical insecticides into ATSB poses risks of environmental contamination and insecticidal resistance (8). Ultraviolet light exploits mosquitoes’ phototaxis towards 365 nm wavelength light, effectively attracting and killing them (9). However, these light sources lack specificity for mosquitoes, often attracting unintended insect species. Commercial lure attractants such as BG-sweetscent and BG-lure, containing compounds that mimic human odors to attract mosquitoes, are typically expensive (10, 11). CO_2_ acts as a potent stimulant of olfactory behavior in mosquitoes, enhancing the attractiveness of lure attractants by two to five times when combined with other attractants (12, 13). Studies have shown that using CO_2_ alone is more effective in capturing *Aedes albopictus* (1.8–2.0 times more) and *Culex pipiens* (15.5 times more) than BG-lure alone when using BG traps (14). Similar findings have been documented by Li et al. (15), Mukabana et al. (16), and Ling and Tan (17). The improved effectiveness, accessibility, and convenience of CO_2_ have led to its integration as a key component in mosquito traps.

In China, CO_2_ was more prevalent in mosquito monitoring traps than in control traps. One plausible explanation for this disparity is the environmental concerns associated with excessive CO_2_ emissions. Monitoring traps require limited operational hours per day, which results in minimal CO_2_ emissions. Conversely, control traps require continuous operation throughout the day to effectively manage mosquito populations, potentially leading to substantial CO_2_ emissions. Consequently, research has predominantly focused on evaluating the impact of CO_2_ on mosquito monitoring, rather than its effects on controlling mosquito density in residential environments.

This study leverages a newly developed breathing mosquito catcher that uses CO_2_ separated from ambient air through innovative nanoporous silicon-based polymer materials. The breathing mosquito catcher will release CO_2_ after harvesting a sufficient amount of CO_2_ to lure the mosquitoes. Compared to existing catchers with CO_2_, such as Mosquito Magnet (MM) and BG traps, this breathing catcher does not produce additional CO_2_ (18). However, the effectiveness of this catcher in controlling mosquitoes in the real world remains unknown. Therefore, in this study, we aimed to identify its effects on mosquito density control through trials in residential settings.

## 2 Methods and materials

### 2.1 Ethics statement

No specific permits were needed for the field studies, as they did not involve endangered or protected species. Residents who supplied electricity for the mosquito catcher gave written informed consent.

### 2.2 Breathing mosquito catcher

**Figure 1:**
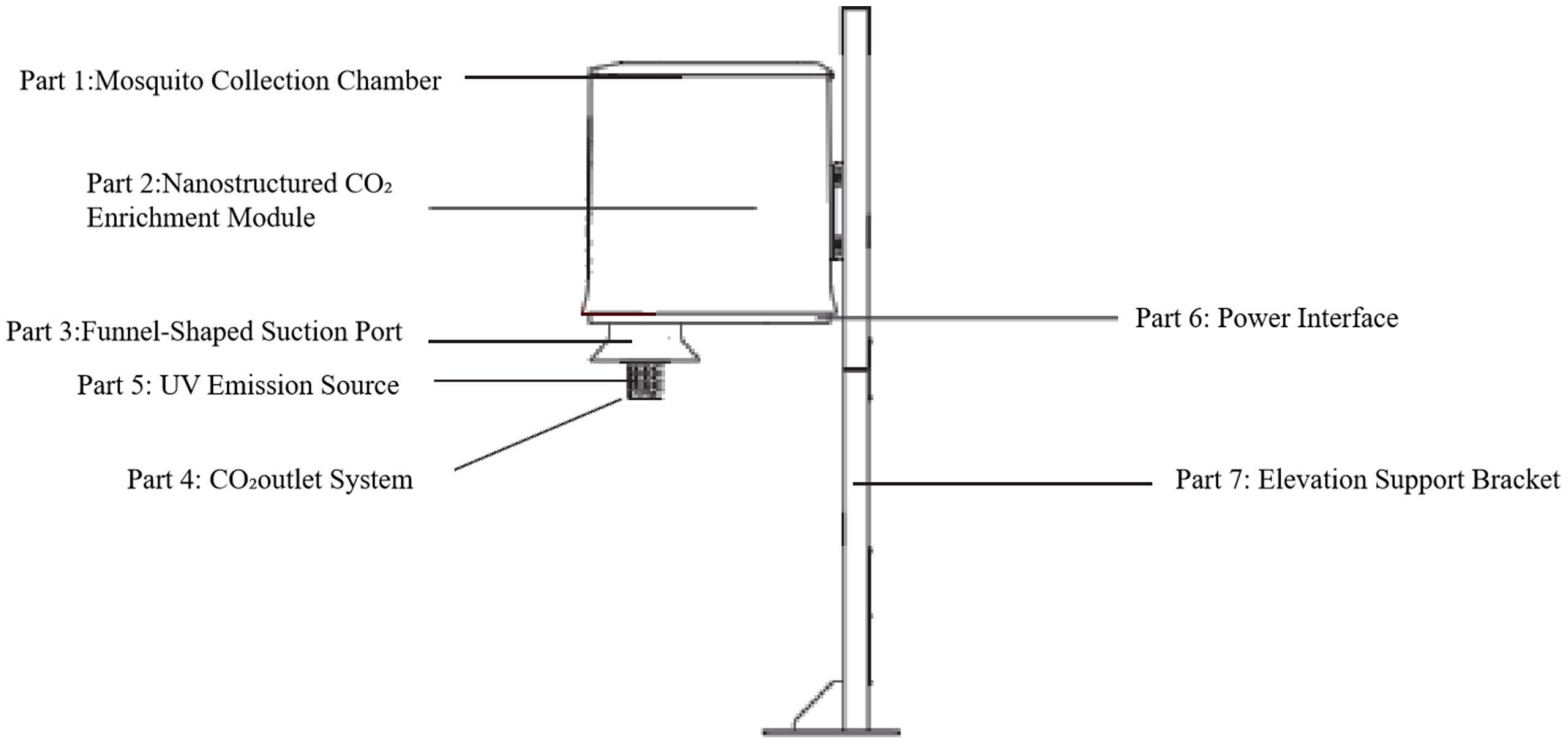
Schematic of the overall design of a breathing mosquito catcher.

A breathing mosquito catcher is shown in Figure 1. It comprises seven functional components, as described below. Electricity was needed, and all the electricity needed was provided by nearby residents after obtaining consent. Part 1 is the Mosquito Collection Chamber used to collect captured mosquitoes from the port in part 3. Part 2 is a Nanostructured CO_2_ Enrichment Module that incorporates a proprietary nano-porous silicon-based polymer matrix (protected under Chinese National Invention Patent Nos. 202021428638.2, PCT/CN2018/098362, PCT/CN2018/098331, 201810489242. X, 201810489846.4, 201710045702.5, and 201710045776.9) engineered to selectively separate and concentrate atmospheric CO_2_. Part 3 is a Funnel-Shaped Suction Port designed with a conical geometry to capture mosquito vectors that lured. Part 4 comprises the CO_2_ Outlet System to regulate and expel enriched CO_2_ at a calibrated flow rate of 20000PPM. Part 5 describes the UV Emission Source. A 365 nm ultraviolet lamp module was integrated to emit shortwave UV radiation. Part 6 comprises the Power Interface. The device was energized via a 130 W-rated power switch, ensuring consistent operational performance. Part 7 is the Elevation Support Bracket. A structural mounting component to suspend the apparatus above ground level.

### 2.3 Study sites

Field trials were conducted in Ningbo City, Zhejiang Province, China, between April and November 2024. Ningbo is situated in the northeast of Zhejiang and has a subtropical monsoon climate, with temperatures ranging from 19 °C to 33 °C and rainfall ranging from 108 mm to 267 mm between April and November 2024. Before the trials began, we selected two villages (GD and XW village, 5.9 km apart) in Ningbo with similar geographic environmental characteristics and preexisting mosquito population densities. The villages were randomly assigned to a control site (GD) or a test site (XW). Trials were divided into two stages: Stage One (April to July) and Stage Two (August to November). Stage One aimed to establish baseline equivalence before trials began. Biweekly mosquito surveillance was conducted from April to July using standardized methodologies. For mosquito larvae surveillance, 50 residential yards were randomly selected at each site, and larval breeding sites were monitored using the larval pipette method. The Container Index (CI) was calculated as follows (19):


$$\begin{array}{l} \mathrm{CI}=\text{number of containers with living mosquito larvae}/\\ \;\;\;\;\;\;\;\;\;\;\;\;\text{number of}\;{\text{ponding containers}}^{*}100\%. \end{array}$$

Adult mosquito populations were stratified according to their diurnal and nocturnal activity patterns. Nocturnally active mosquitoes, such as *Culex pipiens pallens*, were monitored using one CDC light trap operated once overnight at each site, with collections quantified as mosquitoes per trap-night according to the Chinese National Vector Monitoring Program (20). For diurnally active mosquitoes such as *Aedes albopictus*. One BG trap was deployed at each site for half an hour of monitoring between 17:00 and 18:00 h, with sampling intensity standardized as mosquitoes per trap hour. Mosquito monitoring between GD and XW villages was conducted on the same day to minimize mosquito density differences caused by temperature and humidity variations on different dates. Throughout the trials (before and after), the monitoring locations where the CDC light traps and BG traps were placed were fixed to avoid mosquito density bias caused by different monitoring positions. After counting the numbers and identifying the species of mosquitoes collected using the CDC light traps and BG traps, the mosquitoes were released back into the place where they were captured to avoid density fluctuations caused by monitoring.

### 2.4 Study design

**Figure 2:**
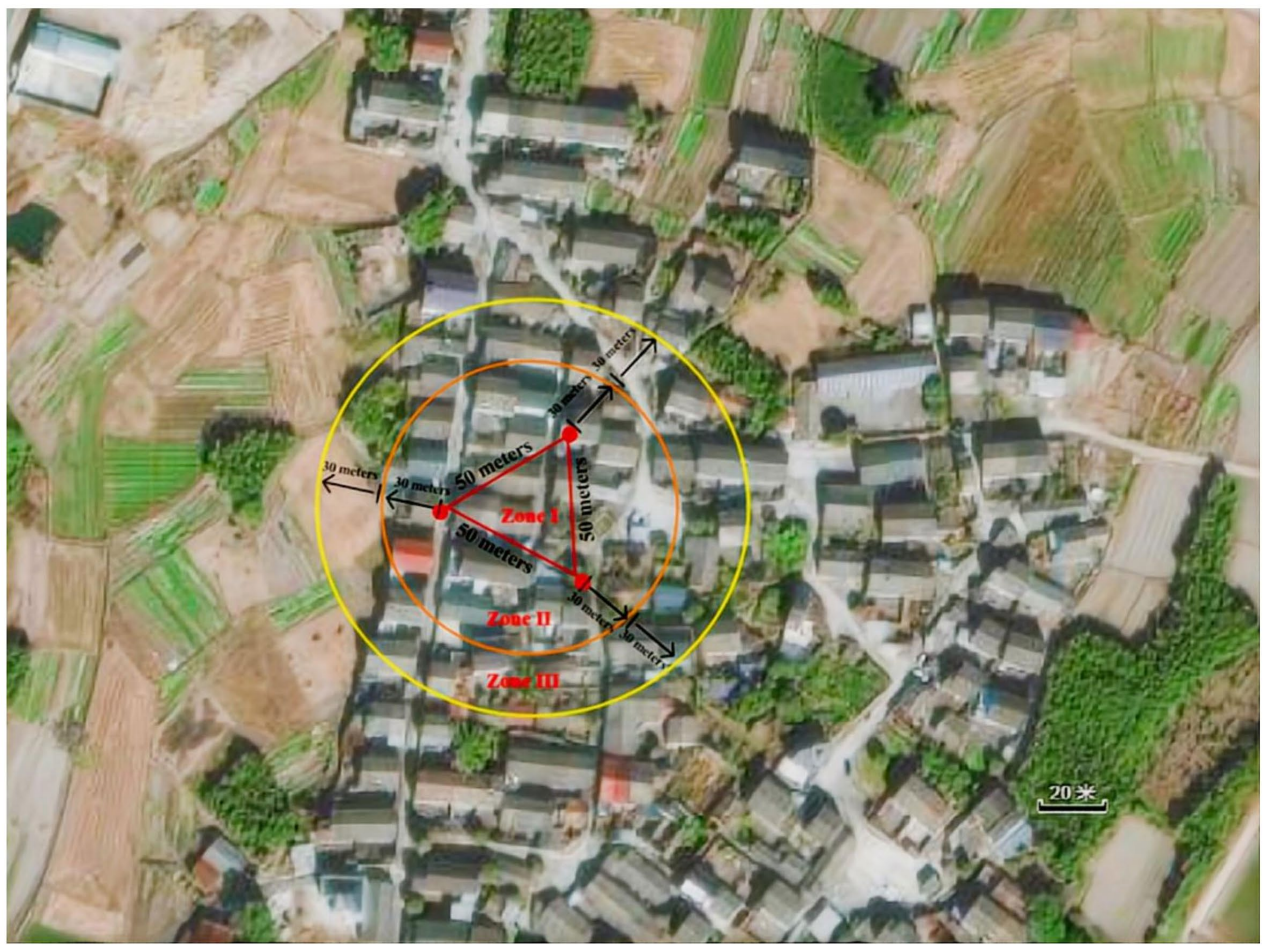
Geographical distribution of Zones I–III in XW village.

Three breathing mosquito catches were set up in XW village residential yards, spaced 50 m apart in an equilateral triangle configuration (Figure 2). The traps were positioned to avoid direct sunlight, artificial lighting, rain, and wind interference. As shown in Figure 2, geospatial zoning was defined as follows:

Zone I: The enclosed area within the equilateral triangle consists of three catches.
Zone II: A circular area extending 30 m radially from each trap perimeter (excluding Zone I).
Zone III: The outer circular area extends an additional 30 m beyond the boundaries of Zone II, excluding Zones I and II.

GD Village was designated as a control group, and no mosquito control measures were performed. After the trials began, larval and adult mosquitoes were monitored every 2 weeks (Stage Two: August–November 2024) in GD village and each zone in XW village using the larval pipette method (CI), CDC light traps, and BG traps. The sampling method was the same as that used before the trials from April to July. When monitoring larval mosquitoes in XW village after the trials, all breeding sites in each zone were monitored because of limited residential yards, whereas in GD village, 50 residential yards were monitored half-monthly. During post-trial monitoring of adult mosquitoes in XW village, nocturnal mosquitoes were surveyed using CDC light traps. Three traps were placed across the three zones, with one trap per zone, and monitoring was conducted overnight on three consecutive “days” under similar climatic conditions. All mosquitoes captured the night before were released after counting and identifying the species to reduce mosquito density fluctuations caused by monitoring. For monitoring diurnal mosquitoes, three BG traps were set across three zones of XW village. Monitoring was conducted over three consecutive “days” under similar climatic conditions, with one CDC light trap placed in each zone for a 30-min sampling period per day(between 17:00 and 18:00 h). All mosquitoes captured the day before were released after counting the numbers and identifying the species. In GD village, the adult mosquito monitoring method and frequency were the same as before the trials.

### 2.5 Statistical analysis

The Statistical Package for the Social Sciences (SPSS, version 23.0) was utilized for conducting the statistical analyses. Nonparametric tests of two independent samples compared larval and adult mosquito densities between GD and XW villages. Generalized linear mixed models (GLMMs) were used to evaluate differences in larval and adult mosquito density among various test groups in GD and XW villages and different zones in XW village during the trials, employing negative binomial regression. Mosquito larvae density served as the dependent variable, with “site” (different villages/zones: control group, Zone I, Zone II, and Zone III), average temperature and precipitation during the week prior to larval monitoring (data from China Meteorological Administration, CMA) included as fixed independent variables. Adult mosquito density was also treated as a dependent variable, with “site” (different villages/zones: control group, Zone I, Zone II, and Zone III), temperature, and humidity on the day (data from CMA) of mosquito monitoring as fixed independent variables. “Days” and “traps ID” were used as random independent variables. Means and standard errors were estimated using GLMMs. Statistical significance was set at *p* < 0.05.

The rates of decrease and standard decrease in mosquito density (adult and larval mosquitoes) were calculated for the GD village and each zone in the XW village before and after the trials (8).

The rate of decrease in adult mosquito density = (average density of adult mosquitoes before trials–average density of adult mosquitoes after trials)/ average density of adult mosquitoes before trials × 100%.

The standard decrease rate of adult mosquito density = (the decrease rate of adult mosquito density in the test group, the rate of decrease in adult mosquito density in the control group)/ (1−the decrease rate of adult mosquito density in the control group) × 100.

The rate of decrease in mosquito larval density was calculated as (average density of mosquito larvae before trials: average density of mosquito larvae after trials) / average density of mosquito larvae before trials × 100%.

The standard decrease rate of mosquito larval density = (the decrease rate of mosquito larval density in the test group−the decrease rate of mosquito larval density in the control group)/(1−the decrease rate of mosquito larval density in the control group) × 100.

## 3 Results

### 3.1 General information

From April to July, larval and adult mosquito densities were monitored twice a month in GD and XW villages to assess baseline comparability between the two sites. The median larval mosquito densities were 24.36 (interquartile range (IQR): 9.93, 37.28) in GD and 29.79 (IQR: 15.51, 33.99) in XW. Median adult mosquito densities were 7.00 (IQR: 3.50, 7.75) and 9.00(IQR: 3.25, 14.00) mosquitoes per trap night using CDC light traps, and 4.00 (IQR: 0.00, 17.00) and 12.00(IQR: 5.00, 27.00) mosquitoes per trap hour using BG traps, in GD and XW villages, respectively (Tables 1–3). No statistically significant differences were observed in larval or adult mosquito densities between the two villages before trials (larval: U = 35, *p* = 0.798; adults—CDC light trap U = 41, *p* = 0.442; BG trap U = 43, *p* = 0.279).

**Table 1:** Mosquito larvae densities (CI) in GD village and different zones in XW village before and after trials.

Field trials	Month	GD village (control group)	XW village(test group)		
			Zone I	Zone II	Zone III
Stage one (before trials)	Early April	0.00	2.38		
	Late April	8.33	15.38		
	Early May	14.71	15.91		
	Late May	32.43	35.00		
	Early June	40.63	33.33		
	Late June	38.89	29.27		
	Early July	28.13	34.21		
	Late July	20.59	30.30		
Stage two (after trials)	Early August	19.44	27.78	17.39	28.00
	Late August	25.00	18.75	21.05	23.08
	Early September	79.63	23.81	28.00	33.33
	Late September	29.41	25.00	25.00	30.30
	Early October	32.56	25.00	38.10	32.14
	Late October	27.27	13.33	12.00	17.24
	Early November	15.38	9.09	5.00	7.69
	Late November	3.23	0.00	0.00	3.70
The decrease rates of mosquito larvae density (%)		−44.01	22.08	23.57	8.17
The standard decrease rates of mosquito larvae density (%)		/	45.90	46.93	36.24

**Table 2:** Differences in mosquito density monitored among different sites.

Sites		Larval mosquito density				Adult mosquito density							
						CDC light trap				BG trap			
		Estimate	SE	*t*	*p*	Estimate	SE	*t*	*p*	Estimate	SE	*t*	*p*
XW village	Zone I	−11.145	4.651	−2.396	0.026^*^	−0.655	0.232	−2.822	0.010^*^	−0.659	0.225	−2.931	0.008^*^
	Zone II	−10.672	4.651	−2.295	0.032^*^	−0.424	0.216	−1.966	0.063	−0.187	0.234	−0.798	0.434
	Zone III	−7.055	4.651	−1.517	0.144	0.001	0.192	0.001	0.990	0.109	0.222	0.491	0.629
GD village^a^		0	/	/	/	0	/	/	/	0	/	/	/

Mean ± SE differences in the least squares means associated with the mixed linear models for the mosquito among different sites (three zones in test village and control village). Estimate: differences in the least squares means. SE, standard error; *t*, *t*-value; *p*, *p* value.

a GD village was selected as the baseline.

^*^Significant differences were found.

**Table 3:** Density of mosquito adults in GD village and different zones in XW village before and after trials measured using the method of the CDC light trap (mosquitoes per trap night).

Field trials	Month	GD village	XW village		
			Zone I	Zone II	Zone III
Stage one (before trials)	Early April	0	1		
	Late April	5	7		
	Early May	8	14		
	Late May	7	15		
	Early June	13	14		
	Late June	7	11		
	Early July	7	4		
	Late July	3	3		
Stage two (after trials)	Early August	4	3	0	3
	Late August	5	3	1	4
	Early September	11	4	5	11
	Late September	16	5	11	15
	Early October	11	6	10	11
	Late October	4	5	6	6
	Early November	2	2	2	4
	Late November	1	0	0	0
The decrease rates of mosquito adult density (%)		−19.00	59.42	49.28	21.74
The standard decrease rates of mosquito adult density (%)		/	65.91	57.39	38.22

### 3.2 Effects of a breathing mosquito catcher on controlling mosquito larvae

After the field trials commenced, natural fluctuations in larval mosquitoes were noted in GD Village, indicating a decrease in mosquito larval density of 44.01%. In different zones (Zones I to III) of XW village, a median of 21.28 (IQR: 10.15, 25.00; 95% confidence intervals, 95% CI: 9.77, 25.92), 19.22 (IQR: 6.75, 27.25; 95% CI: 7.88, 28.75), and 25.54 (IQR: 10.08, 31.68; 95% CI: 12.46, 31.41) larval mosquito densities (CI) per half-month were observed, with corresponding decreases in mosquito larvae density of 22.08, 23.57, and 8.17%, respectively (Table 1). Post-adjustment for the control group, the standardized decrease rates of mosquito larval density were 45.90, 46.93, and 36.24%. Zones I and II in XW Village exhibited significantly lower larval densities compared to GD village post-trials (Table 2).

### 3.3 Effects of a breathing mosquito catcher on adult mosquito control (monitored using CDC light traps)

**Figure 3:**
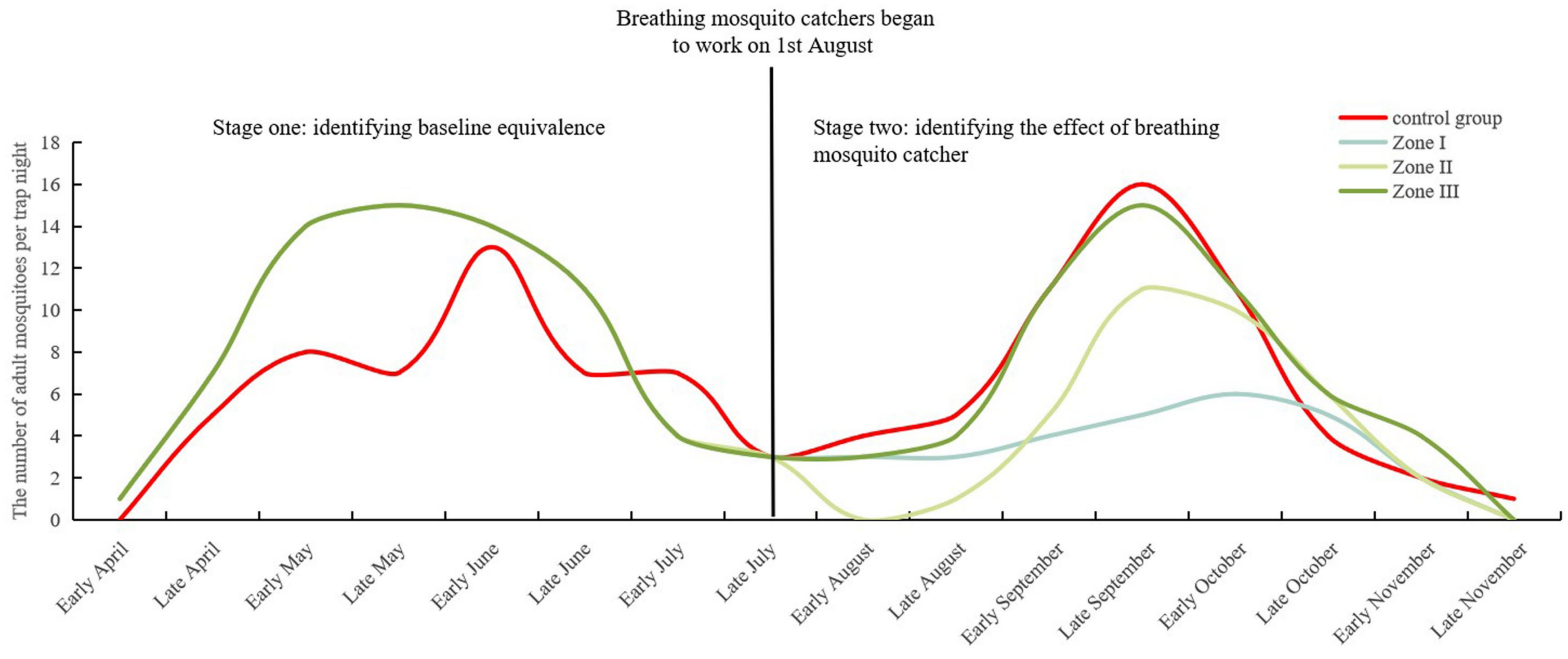
Density changes in adult mosquitoes monitored using the CDC light trap in GD (control group) and different zones (Zone I to III) of XW village before and after trials.

A total of 171 adult mosquitoes were captured using CDC light traps in the GD (54 mosquitoes) and XW (117 mosquitoes) villages post-trials. Five species were identified, with *Culex pipiens pallens* being the most abundant (90.06%), followed by *Anopheles* sinensis (3.51%) and *Culex tritaeniorhynchus* (2.92%). Natural fluctuations were observed in adult mosquitoes in GD village, with a decrease in the rate of mosquito larvae density of −19.00% (Table 3). A median of 3.50 (IQR: 2.25, 5.00; 95% CI: 1.89, 5.11), 3.50 (IQR: 0.25, 9.00; 95% CI: 0.72, 8.03), and 5.00 (IQR: 3.25, 11.00; 95% CI: 2.52, 10.98) adult mosquitoes per trap night were monitored in different zones (Zone I–III) of XW village, with decrease rates of mosquito larvae density at 59.42, 49.28, and 21.74%, respectively (Figure 3). After correction for the control group, the standard decrease rates of the mosquito larval density were 65.91, 57.39, and 38.22%, respectively. Compared to GD village, Zone I in XW village showed significantly lower larval densities after the trials (Table 2).

### 3.4 Effects of a breathing mosquito catcher on adult mosquito control (monitored using BG traps)

A total of 100 adult mosquitoes were captured using the BG trap in GD (29 mosquitoes) and XW (71 mosquitoes) villages post-trials. Two species were identified: *Aedes albopictus* (85.00%) was the most abundant, followed by *Culex pipiens pallens* (15.00%). Following the trial commencement, a median of 6.00 (IQR: 1.00, 11.00; 95% CI: 1.19, 13.30), 5.00 (IQR: 0.00, 6.00; 95% CI: 1.00, 6.49), 5.00 (IQR: 2.00, 9.50; 95% CI:1.44, 10.56), 6.00 (IQR: 2.50, 12.00; 95% CI: 2.00, 13.99) of adult mosquitoes per trap hour were monitored in GD village and different zones (Zone I to III) of XW village by BG traps, with the decrease rate of mosquito adult density −81.25, 51.61, 22.58, −3.23%, respectively (Table 4). After correction for the control group, the standard decrease rates of adult mosquito density were 73.30, 57.29, and 43.05%. Compared with GD village, Zone I in XW village showed significantly lower adult mosquito densities after the trials (Table 2).

**Table 4:** The density of mosquito adults in GD village and different zones in XW village before and after trials using the method of BG trap (mosquitoes per trap hour).

Field trials	Month	GD village	XW village		
			Zone I	Zone II	Zone III
Stage one (before trials)	Early April	0	0		
	Late April	0	2		
	Early May	2	6		
	Late May	6	6		
	Early June	12	18		
	Late June	10	20		
	Early July	2	4		
	Late July	0	6		
Stage two (after trials)	Early August	4	0	2	4
	Late August	4	4	2	8
	Early September	8	8	8	4
	Late September	22	6	10	12
	Early October	12	6	16	22
	Late October	8	6	8	12
	Early November	0	0	2	2
	Late November	0	0	0	0
The decrease rates of mosquito adult density (%)		−81.25	51.61	22.58	−3.23
The standard decrease rates of mosquito adult density (%)		/	73.30	57.29	43.05

## 4 Discussion

Carbon dioxide is frequently used as an attractant for mosquito trapping. In China, a new mosquito breathing catcher utilizing CO_2_ (extracted and concentrated from the surrounding air using nano-porous silicon-based polymer materials) has been developed to control mosquitoes within residential settings. This research assessed the impact of this mosquito trap on mosquito populations in field trials. The findings showed that the trap significantly decreased both larval and adult mosquito densities within certain regions.

Mosquitoes act as vectors for various arboviral diseases, which pose significant public health and socioeconomic challenges in tropical and temperate regions (13). Reduction in mosquito populations would reduce human-vector contact rates then lower the risk of transmission for arboviruses. Thus, effective control strategies for mosquito-borne diseases rely on robust mosquito surveillance and control. This study assessed the efficacy of a newly developed trap in reducing mosquito populations in residential areas. Prior to the experiments, we conducted a four-month longitudinal surveillance in GD and XW villages to establish similar baseline mosquito densities in the test and control groups. The analysis revealed no significant differences in larval density (*p* = 0.798) or adult mosquito densities (CDC light trap, *p* = 0.442; BG trap, *p* = 0.279) between the two groups. These baseline data confirmed the comparability of the groups and supported the subsequent evaluations of vector control interventions.

This study emphasizes evaluating mosquito population reduction rates as a more direct indicator for assessing interventions against mosquito-borne diseases rather than relying solely on trap-specific capture counts. A significant decline in larval and adult mosquito densities was observed across three zones (I–III) in XW village after the trials began, with standardized mosquito decrease rates reaching 46.93% (larval, Zone II), 65.91% (CDC light trap, Zone I), and 73.30% (BG trap, Zone I). These results are consistent with Wang et al. (21) and Pan et al. (22), who demonstrated similar efficacy using UV light- and CO_2_-baited mosquito control devices. Effective mosquito control was also noted in larval and adult mosquitoes between Zone I in XW and GD villages (control group), indicating that the effective radius for larval and adult mosquito control was less than 30 m. Therefore, in future applications, the distance between catches should not exceed 60 m (twice the effective radius of one catch).

Circadian activity patterns in mosquitoes can be significantly influenced by photoperiodic cycles, temperature, humidity conditions, and species-specific genetic regulatory mechanisms, leading to distinct temporal peaks in host-seeking behavior across various mosquito taxa (23). For example, *Aedes albopictus* shows bimodal activity peaks between 6:00 and 8:00 and 16:00 and 18:00, while *Culex pipiens pallens* and *Culex tritaeniorhynchus* display nocturnal activity peaks between 21:00–24:00 and 23:00–1:00 am, respectively (12). In Zhejiang Province, *Aedes* and *Culex* were the predominant mosquito species (24). Therefore, in field trials, two different traps were used to monitor adult mosquitoes, with CDC light traps tracking nocturnal mosquito populations and BG traps monitoring diurnally active species. The results showed that 92.98% of mosquitoes captured by CDC light traps were identified as *Culex* spp., whereas 85.00% of the BG trap catches belonged to *Aedes* spp. These results align with previous entomological surveys by Zhang et al. (24) and Duan et al. (25), indicating that *Culex pipiens* pallens and *Aedes albopictus* were the most prevalent mosquito species in Zhejiang Province. Importantly, reductions in population density associated with interventions were observed in both *Aedes* and *Culex* populations within 30 m mentioned above, indicating the effectiveness of the trap across dominant vector species in Zhejiang with varying circadian behaviors.

CO_2_ activates olfactory-driven host-seeking behavior in mosquitoes, serving as a potent attractant (1). Prior research has shown the impact of CO_2_ on mosquito surveillance and control, particularly in vector mosquitoes (26, 27). Excessive anthropogenic CO_2_ emissions significantly contribute to climate change, leading to the greenhouse effects and disrupting soil carbon dynamics (28, 29). Elevated atmospheric CO_2_ levels may indirectly heighten public health risks by worsening respiratory and cardiovascular conditions through climate-related events like extreme weather and increased air pollution (30, 31). These environmental and health concerns highlight the need for mosquito control strategies to reduce additional CO_2_ emissions. To tackle this issue, we introduce a novel mosquito breathing catcher that combines biomimetic engineering principles with advanced materials science (Chinese National Invention Patents 202021428638.2, PCT/CN2018/098362, PCT/CN2018/098331, 201,810,489,242. X, 201810489846.4, 201710045702.5, and 201710045776.9). This catcher utilizes a closed-loop CO_2_ capture and release system to extract metabolic CO_2_ directly from the surrounding air. By enhancing atmospheric CO_2_ levels without introducing external sources, the catcher maintains operational efficiency while significantly reducing its carbon footprint. This environmentally friendly innovation demonstrates equal attractiveness to both diurnal and nocturnal mosquito species within certain regional ranges, providing a sustainable control solution for residential areas.

Given the primary aim of the newly developed mosquito trap to reduce mosquito density, our experimental design prioritized assessing its real-world efficacy in this regard. We have yet to conduct comparative studies on its horizontal mosquito capture capabilities against other traps like MM and BG traps. Secondly, this study is geographically narrow, limited to two villages in Ningbo, and the intervention period covers only several months, which may not capture longer-term seasonal or interannual fluctuations in vector populations, thus extension should be cautious when relating to other ecological/climatic contexts and other months. On the other hand, we did not identify non-target insect species captured by this breathing mosquito catcher, which resulted in an inability to evaluate non-target insect impacts or long-term ecological consequences using this newly developed catch. Further detailed research is needed to evaluate the application potential of this breathing CO_2_ capture method.

## 5 Conclusion

Breathing mosquito catchers could effectively control larval and adult mosquitoes within certain regional ranges surrounding residential areas. Without releasing additional CO_2_, this could be a more environmentally sustainable option than conventional attractant-based devices that rely on dry ice or pressurized cylinders. However, the effect was most pronounced within approximately 30 m of the traps, and wider deployment of traps would require careful planning of trap density.

## Data Availability

The original contributions presented in the study are included in the article/supplementary material, further inquiries can be directed to the corresponding author.
